# NETosis of Peripheral Neutrophils Isolated From Dairy Cows Fed Olive Pomace

**DOI:** 10.3389/fvets.2021.626314

**Published:** 2021-04-29

**Authors:** Maria Giovanna Ciliberti, Marzia Albenzio, Salvatore Claps, Antonella Santillo, Rosaria Marino, Mariangela Caroprese

**Affiliations:** ^1^Department of Agriculture, Food, Natural Resources, and Engineering, University of Foggia, Foggia, Italy; ^2^Council for Agricultural Research and Economics—Research Centre for Animal Production and Aquaculture, Bella Muro, Italy

**Keywords:** ruminants, olive pomace, immune innate system, by-products, inflammation

## Abstract

Neutrophils represent primary mobile phagocytes recruited to the site of infection, and their functions are essential to enhance animals' health performance. Neutrophils have an essential role in innate immunity and are able to kill the pathogens *via* the synthesis of neutrophil extracellular traps (NETs). The objective of the present work was the study of the *in vitro* NETosis of peripheral neutrophils isolated from dairy cows supplemented with olive pomace. Dairy cows (*n* = 16) balanced for parity (3.67 ± 1.5 for CON, 3.67 ± 1.9 for OP), milk yield (24.3 ± 4.5 kg d^−1^for CON and 24.9 ± 1.7 kg d^−1^ for OP), the number of days in milk (109 ± 83.5 for CON and 196 ± 51 for OP), and body weight (647 ± 44.3 kg for CON and 675 ± 70.7 kg for OP) were divided into two experimental groups fed with a control diet (CON) and supplemented with 6% of olive pomace (OP). Peripheral blood neutrophils were isolated and stimulated *in vitro* with phorbol-myristate-acetate (PMA) as a marker for activation and reactivity of the neutrophils. After isolation, both the viability and CD11b expression were analyzed by flow cytometry. Both NETosis by neutrophil elastase-DNA complex system and myeloperoxidase (MPO) activity were evaluated by ELISA. The specific antibodies against MPO and citrullination of Histone-H1 were used for investigating NETosis by immunofluorescence microscopy. The neutrophil elastase-DNA complexes produced during NETosis and MPO activity of neutrophil extracts were affected by OP supplementation. Furthermore, results from immunofluorescence analysis of NETosis depicted a similar result found by ELISA showing a higher expression of MPO and citrullination of Histone-H1 in OP than the CON neutrophils. In addition, all data showed that the OP diet resulted in a better response of neutrophils to PMA stimulation than the CON diet, which did not support the neutrophils' responses to PMA stimulation. Our results demonstrated that OP supplementation can enhance the neutrophil function in dairy cows leading to udder defense and inflammation response especially when an immunosuppression state can occur.

## Introduction

Neutrophils are considered the frontline defenders of innate immunity recruited for the suppression and the clearance of invading pathogens to an inflammatory site. Different defense strategies can be initiated by neutrophils, among which the antimicrobial mechanism involved in neutrophil extracellular traps (NETs) formation (1, 2). This mechanism consists of processed chromatin or DNA decorated with histones characterized by specific cytoplasmic proteins from the neutrophilic granules that are extruded into the extracellular environment when neutrophils undergo NETosis, a dynamic cell death program of neutrophils responsible for NET formation (3). The pathogens get trapped into the physical barrier of NETs and can be disarmed and killed extracellularly (4, 5). DNA is a major structural component of NETs. Using immunofluorescence analysis, it emerges that NETs contain components with bactericidal activities from primary granules (6, 7) such as neutrophil elastase (NE), cathepsin G, and myeloperoxidase (MPO), as well as proteins from secondary and tertiary granules, such as lactoferrin and gelatinase. NET formation is considered a beneficial neutrophil response with the main aim of controlling the invading of infective microorganisms (8); however, this dysregulation can be harmful to the host (9). The antimicrobial function of neutrophils has long been considered the unique role of these cells, defined as fixed pre-programmed immune cells; however, in the last decade, their extensive plasticity functions have been proposed (10–13).

Indeed, neutrophils have emerged as important mediators between the innate and adaptive immune systems, as demonstrated by a number of cytokines and chemokines secreted that affect the immune response during an inflammatory process (14, 15). Moreover, they have a central role in tissue remodeling and in homeostasis maintenance by disposing of apoptotic cells and phagocytosing foreign particles (16, 17). The understanding of the new emerging role of neutrophils, at the crossroads of innate and adaptive immunity, could be strategical when animals are exposed to stress condition, such as transition period or heat stress, being more vulnerable to immunosuppression due to change in the metabolic, physiological, and immunological state. It has been demonstrated that immediately post-calving, about 30–50% of the cows experienced health disorders (18). In particular, the negative energy balance (NEB) status around calving is considered a major contributing factor to periparturient immune dysfunction caused also by the increased plasma non-esterified fatty acid (NEFA) concentrations due to the lipid mobilization (19). Furthermore, in parturient cows, reduced neutrophil adhesion, migration, and phagocytosis-induced respiratory burst activities are observed (20). A diagnostic tool that measures the total number of blood neutrophils, the proportion of immature neutrophils along with an alteration in the genes involved in neutrophil adhesion, chemotaxis, and phagocytosis on the day of calving succeeded in monitoring the health status of dairy cattle (21–24).

It is worth noting that a well-balanced diet is strictly required for healthy and highly productive cows. Indeed, dietary supplementation with antioxidants, including vitamins, and trace minerals, may represent a very attractive strategy for boosting the immunity of dairy cattle during immunosuppressive conditions. A latest sustainable alternative has been represented by the utilization of byproducts in animal nutrition, which concomitantly decrease the feed costs and valorize a waste biomass; this last concept is a key point in the European Union's 2020 Environment Action Programme about waste management, recycling, and reuse (25). Olive pomace has been tested in dairy ruminant species, without any significant contribution to milk production (26–29) and growth rate (30–32). However, stoned olive cake results in a significant increase in the unsaturated fatty acids/saturated fatty acids (UFAs/SFAs) ratio and in the oleic acid (OA) content and a decrease in atherogenic and thrombogenic indices in ewes' milk (33, 34). The role of fatty acids n-3 supplementation into the diet was found out to be capable of modulating the functional properties of lymphocytes and mononuclear cells in transition ruminants (35, 36). Moreover, supplementing cows during the transition period with n-6 fatty acids resulted in better acute phase responses and enhanced neutrophil function (37).

At present, no studies evaluated the role of dietary olive pomace (OP) supplementation of dairy cows on neutrophil function. Therefore, the aim of this study was to assess the *ex vivo* antimicrobial activity of NETosis and myeloperoxidase activity of peripheral neutrophils isolated by cows fed with olive pomace.

## Materials and Methods

### Animals

Sixteen dairy cows were assigned to two isoenergetic and isonitrogenous feeding regimens.

The control group (CON) was fed with 8 kg/cow daily of pelleted concentrate, whereas the OP group received 8 kg/cow daily of pelleted concentrate with the inclusion of 6% of olive pomace. The average parity of the cows was 3.67 ± 1.5 for CON and 3.67 ± 1.9 for OP (mean ± standard deviation), the milk yield was 24.3 ± 4.5 kg d^−1^ for CON and 24.9 ± 1. 7 kg d^−1^ for OP, the number of days in milk was 109 ± 83.5 for CON and 196 ± 51 for OP, and the body weight was 647 ± 44.3 kg for CON and 675 ± 70.7 kg for OP. All groups were individually fed twice daily and received 12 kg/cow daily of oat hay and were kept in free-stall housing condition. The experiment lasted for 30 days. Water was offered *ad libitum*. Dry matter (DM), ash, ether extract (EE), and crude protein (CP) were determined as described by AOAC International (38). Briefly, the CON diet contained 16.1% CP, 3.1% EE, and 6.80% of ash, while the OP diet contained 15.4% CP, 3.19% ether extract, and 7.02% of ash (calculated on a DM basis, 93.48% for CON group and 93.52% of OP). The palatability of the diet was assessed by the evaluation of the refusal and the study of feces consistency; both parameters were not affected by the diet. Milk yield and composition were determined throughout the experiment; on average, milk yield was 20.97 kg/day for the OP group and 20.85 kg/day for the CON group. Furthermore, milk composition was, on average, characterized by 3.23 ± 0.48 of fat, 2.98 ± 0.29 of protein, and 2.31 ± 0.19 of casein content in the OP group, whereas by 3.07 ± 0.51 of fat, 3.04 ± 0.25 of protein, and 2.33 ± 0.18 of casein content in the CON group.

A representative sample of feed supplementation was collected for FA analysis of feed by the determination of methyl esters according to O'Fallon et al. (39). Briefly, 1 g of each sample was pipetted into a screw-cap (16 × 25 mm) reaction tube. Into each tube, 1.0 mL of the C13:0 internal standard (0.5 mg of C13:0/mL of methanol), 0.7 mL of KOH, and 5.3 mL of methanol were added during incubation at 55°C for 1.5 h, and the tubes were inverted to mix for 5 s every 20 min. After cooling the tubes in a cold water bath, 3 mL of hexane was added to each tube and mixed by vortex for 5 min. The tubes were centrifuged at room temperature for 5 min at 500 × g; then 1 mL of supernatant was taken from each tube, transferred into vials, and stored at −20°C for analysis by GC. Fatty acid profiles were quantified using a GC (6890N; Agilent Technologies, Santa Clara, CA) equipped with a flame-ionization detector. Helium was the carrier gas, the gas flow rate was 0.8 mL/min, and the column head pressure was 175 kPa. The oven temperature (40) was initially held at 70°C for 4 min and then programmed to increase at 13°C/min to 175°C and then held isothermally for 45 min. The column used was a capillary column (HP88; 100 m × 0.24 mm i.d., 0.20-μm film thickness, Agilent Technologies). Concentrations of FAME were analyzed utilizing a calibration curve with a mixture of standards of 50 FA (GLC Reference standard 674, Nu-Chek Prep Inc., Elysian, MN) with added CLA standards: C18:2 trans-8, cis-10; C18:2 cis-9, trans-11; C18:2 cis-11, trans-13; C18:2 trans-9, cis-11; C18:2 cis-8, cis-10; C18:2 cis-9, cis-11; C18:2 trans-10, cis-12; C18:2 trans-8, trans-10; C18:2 trans-9, trans-11; C18:2 trans-10, trans-12; and C18:2 trans-11, trans-13 (GLC Reference standard UC-59M, Nu-Chek Prep Inc.). All procedures were conducted according to the guidelines of EU Directive 2010/63/EU (41) on the protection of animals used for experimental and other scientific purposes. Dairy cows were healthy, and their conditions were carefully examined by veterinarians throughout the trial to exclude the presence of signs of diseases.

### Blood Samples and Peripheral Neutrophil Isolation

Blood samples from animals at 30 days of the experiment were collected from the jugular vein into sterile vacuum tubes containing heparin (Becton Dickinson). All experiments were performed using peripheral neutrophils obtained from 16 dairy cows (*n* = 8 for the OP group and *n* = 8 for the CON group).

Briefly, whole blood was diluted 1:1 with cold PBS and slowly layered on the Histopaque®-1077 solution (10 mL). The tubes were centrifuged at 1,500 g for 30 min at 20°C; the buffy coat containing the PBMCs was discarded. The remaining cells were treated with lysis buffer represented by cold NaCl (0.2% in PBS), according to Baien et al. (42), with some modifications, in order to remove red blood cells. After 30 s of gentling inversion mixing, 20 mL of cold NaCl (1.6% in PBS) was added followed by gentling mixing with inversion for 5 s and centrifuged for 8 min at 500 g at room temperature (RT). The lysis step was repeated two times or until a clear neutrophil pellet was obtained. After the final wash, the pellet was resuspended in 1 mL of RPMI 1640 without phenol red, and neutrophil concentrations were measured using the trypan blue exclusion method on a Countess™ II Automated Cell Counter (Thermo Fisher).

**Figure 1:**
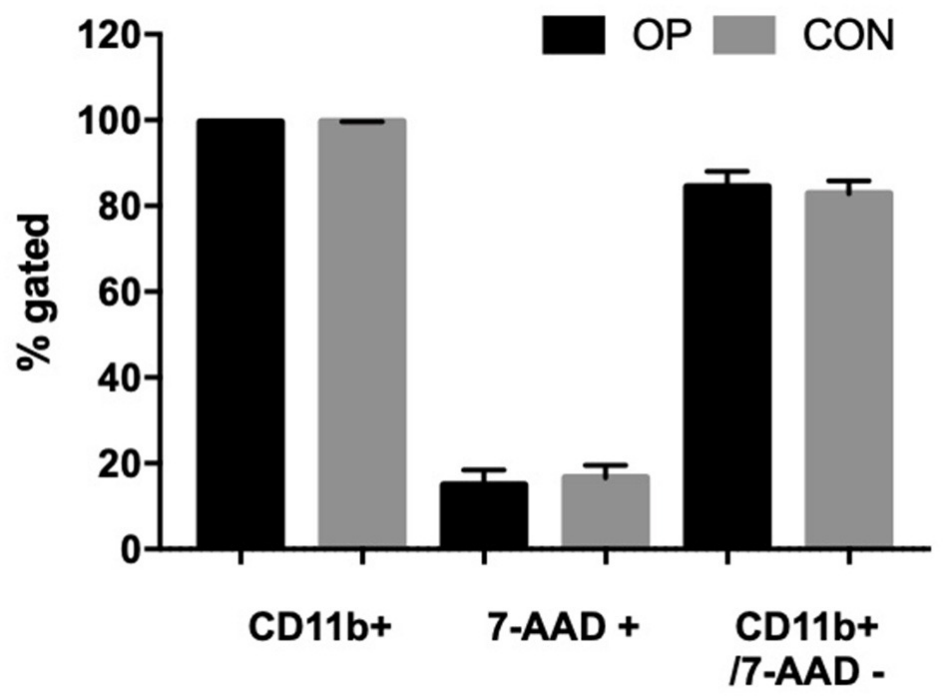
Percentages of viable peripheral neutrophils after isolation. Cells were stained with CD11b as the neutrophil marker and 7-AAD as the viable dye. CD11b and 7-AAD fluorescence was collected in the BL1 (530/30) and BL3 (695/40) channels, respectively.

Immediately after isolation, 5 × 10^5^ cells/100 μl were stained with 5 μl of FITC-labeled antibody against CD11b and incubated for 30 min in the dark at room temperature. Cells were washed twice with 1 × PBS and centrifuged at 200 × g 4°C for 10 min. Finally, the pellet was resuspended in 200 μl of PBS, and 5 μl of 7-AAD Viability Staining Solution (Exbio) was added and incubated for 10 min at room temperature in the dark. The positive dead control cells were represented by cells treated with 100 μl buffer containing 0.2% Triton X-100 (Sigma Aldrich) and 2% BSA in PBS (Sigma Aldrich). Unstained cells were used as 7-AAD and CD11b negative control. Threshold was adjusted to unstained cells to remove background (i.e., noise). The acquisition volume was set to 50 μl (total draw volume 100 μl), the acquisition speed was set to 100 μl/min, and a total of 10,000 events were recorded. CD11b and 7-AAD fluorescence was collected in the BL1 (530/30) and BL3 (695/40) channels, respectively. Cells were analyzed by Attune NxT Flow Cytometer (Thermo Fisher). For the determination of living and dead cells, gates were set with regards to the dead control and the unstained control (live cells). The positive CD11b cells had about >98% of purity (Figure 1), whereas the viable CD11b^+^ (CD11b^+^/7-AAD^−^) cells had about 84% (Supplementary Figure 1).

### Immunofluorescence Staining of NETs

Isolated neutrophils (5 × 10^5^ cells) from each experimental group (OP and CON) were seeded in 48-well plates (Falcon™ tissue culture treated) and allowed to adhere for 30 min at 37°C. Cells were stimulated or not with PMA 50 ng/ml for up to 3 h. After stimulation, supernatants were discarded, and cells were washed twice with PBS and fixed in 4% paraformaldehyde for 15 min at RT. Cells were washed twice with BSA 3%/PBS. For the permeabilization step, 500 μl of Triton-x 0.5% in PBS was added, and the plate was incubated for 20 min at RT. The fixing solution was removed, and cells were washed two times with BSA 3%. Then, the plates were blocked with BSA 3% for 1 h to prevent non-specific binding and washed with PBS. To detect histone and MPO, each well was incubated with 5 μl (400 μl) Histone-H1 Polyclonal Antibody (Invitrogen) and 5 μl of Anti-Human Myeloperoxidase (MPO)-FITC overnight at 4°C. On the next day, the plate was washed twice with BSA 3%/PBS, then 500 μl of Goat anti-Rabbit IgG (H+L) Alexa Fluor plus 594 conjugated (Invitrogen, 1/1,000), as secondary antibody for Histone-H1, and Hoechst 33342 for nuclei detection (5 μg/mL) was added for 1 h at RT. Finally, the plate was washed with PBS and imaged on the Life Technologies EVOS™ XL Imaging System (Milan, Italy). The nucleus or DNA was detected in the DAPI channel, the MPO in the GFP channel, and the Histone-H1 in the Texas red channel.

### Measurement of NE-DNA Complexes Produced by NETosis

Isolated neutrophils (5 × 10^5^ cells) from each experimental group (OP and CON) were seeded in 48-well plates (Falcon™ tissue culture treated) and allowed to adhere for 30 min at 37°C. Cells were stimulated or not with PMA 50 ng/mL for up to 3 h. At 37°C, NE-DNA complexes were measured using a NETosis Assay Kit (Cayman Chemical, Ann Arbor, MI, USA). Briefly, unbound NE in the supernatant was discarded following NET generation. After adding S7 nuclease, the soluble elastase was dissociated from NET-associated DNA and then added to a substrate; this was selectively cleaved by elastase to yield a 4-nitroaniline, which absorbs light at 405 nm.

### Myeloperoxidase Activity Assay

Isolated neutrophils (5 × 10^5^ cells) from each experimental group (OP and CON) were seeded in 48-well plates (Falcon™ tissue culture treated) and allowed to adhere for 30 min at 37°C following stimulation or not with PMA 50 ng/mL for up to 3 h at 37°C as a positive control of NET formation. Neutrophils were lysed by multiple freeze-thaw cycles, and the neutrophil extracts were collected after centrifugation at 12,000 rpm for 15 min at 4°C. The MPO enzyme is responsible for antimicrobial activity and expressed in stimulated neutrophils, where it catalyzes the production of hypoalous acids, such as hypochlorous acid (HOCl), from hydrogen peroxide (H_2_O_2_) and chloride ion (Cl^−^), or other halides. The MPO activity was determined in neutrophil extracts using a commercial kit (Cell Biolabs, USA), according to the manufacturer's instructions. Briefly, the neutrophils were incubated with 1 mM hydrogen peroxide (H_2_O_2_) for 30 min at room temperature. A catalase-containing stop solution and chromogen were added. The absorbance was then measured at 405 nm.

### Statistical Analysis

Data were checked for normality test and analyzed with the MIXED ANOVA model of SAS (43). The model included the fixed effects of *in vitro* treatment (PMA), the feeding strategy (CON and OP), and their interaction. Animals are included in the model as a random effect. The significance of the differences was assessed using Tukey *post-hoc*-test for multiple comparisons, and a *P*-value of < 0.05 was considered statistically significant. *P* < 0.10 was considered a tendency. Data were presented as mean ± SEM. Data on the fatty acids profile of the experimental diet were analyzed using one-way ANOVA of SAS (43). The significance of the differences was assessed by the Tukey-test. Significance was declared at *P* < 0.05. Pearson correlation analysis was performed to correlate the levels of NE-DNA complexes and MPO activity. Immunofluorescence staining of NETs of single cell staining was analyzed using Image J (National Institute of Health, Bethesda, MD, United States) as described previously (44). Area, integrated density, and mean gray value were collected. In order to calculate the corrected total cell fluorescence (CTCF), the mean fluorescence of background was collected using areas without fluorescence adjacent to cells. The equation applied was CTCF = integrated density − (area of selected cell × mean fluorescence of background). Data from immunofluorescence of NETs were analyzed by ANOVA using Turkey's *post-hoc*-test for multiple comparisons using the GraphPad Prism 7 software (GraphPad Software, La Jolla, CA, United States). Results were expressed as mean ± SEM.

## Results

### Fatty Acids Profiles of Experimental Diets

The FA profile of experimental diets is shown in Table 1. Palmitic acid (C16:0), stearic acid (C18:0), and saturated fatty acids (SFA) were higher in the CON diet than in the OP diet. As expected, the most abundant FAs in OP were oleic acid (C18:1 cis9) and linoleic acid (C18:2 c9c12). Moreover, the OP diet resulted in a major proportion of monounsaturated fatty acids (MUFA), P/S, and polyunsaturated fatty acids n6 as compared with the CON diet.

**Table 1:** Fatty acids profile of the CON and OP diets.

	**Diet**^****a****^			***P*-value**
**Fatty acids, g/100 g of FAME**	**CON**	**OP**	**SEM**	
C16:0	40.88	31.47	0.39	0.002
C18:0	4.51	3.95	0.01	0.016
C18:1c9	28.06	32.83	0.16	0.009
C18:2c9c12	12.77	14.10	0.04	0.001
C18:3n3	0.79	0.76	0.01	0.870
SFA	50.32	40.52	0.38	0.002
MUFA	29.76	37.26	0.21	0.001
PUFA	19.92	22.22	0.58	0.058
P/S	0.40	0.55	0.01	0.009
n6	17.90	19.85	0.14	0.006
n3	1.67	1.88	0.11	0.207

a *Diet = CON, control diet based on pelleted concentrate: maize (20%), dehusked soybean meal (16%), wheat bran (15.3%), millings wheat (15%), sunflower seed meal (12%), flaked corn (10%), sugar beet pulp (6%), mineral and vitamin mix (4.7%), and sucrose (1%); OP = experimental diet based on pelleted concentrate with inclusion of 6% of olive pomace: maize (21.14%), millings wheat (20%), dehusked soybean meal (18%), flaked corn (10%), sunflower seed meal (10%), wheat bran (6.7%), olive pomace (6%), sugar beet pulp (3%), mineral and vitamin mix (4.16%), and sucrose (1%). Data were presented as least squares means ± standard error mean*.

### The Levels of NE-DNA Complexes Produced by NETosis

**Figure 2:**
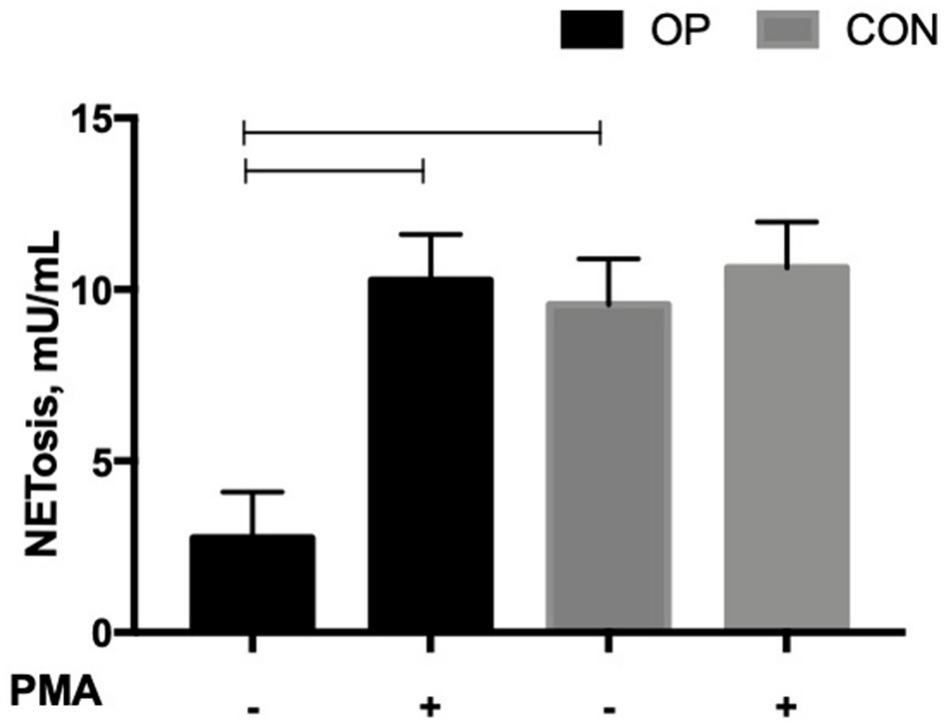
Mean ± SEM of NE-DNA complexes (mU/mL) produced by NETotic neutrophils evaluated using the NETosis Assay Kit. Cells (5 × 10^5^ cells) from each experimental group (OP and CON *n* = 8 for each) were seeded in 48-well plates and allowed to adhere for 30 min at 37°C. Cells were stimulated or not with PMA 50 ng/mL for up to 3 h at 37°C.

The levels of NE-DNA complexes produced by NETotic neutrophils were significantly affected by *in vitro* PMA stimulation (*P* = 0.0311); a tendency for both the feeding strategy (*P* = 0.0683) and the interaction between *in vitro* PMA stimulation and the feeding strategy (*P* = 0.0978) was registered. In Figure 2, the OP NETotic neutrophils *in vitro*, with no stimulation by PMA, showed the lowest level of NE-DNA complexes. The CON NETotic neutrophils did not alter their level of NE-DNA complexes after *in vitro* PMA stimulation (9.55 ± 1.34 vs. 10.63 ± 1.34); on the contrary, OP NETotic neutrophils significantly increased their level of NE-DNA complexes after *in vitro* PMA stimulation (2.75 ± 1.34 vs. 10.27 ± 1.34).

### Intracellular ROS Generation Controlled by Myeloperoxidase Activity

**Figure 3:**
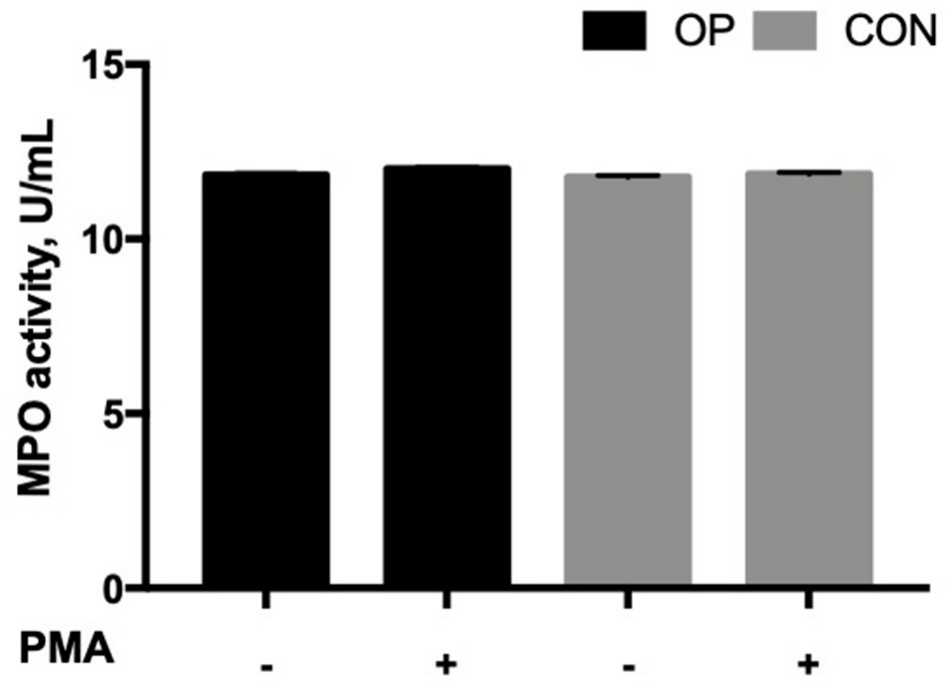
Mean ± SEM of MPO activity (U/mL) of neutrophils. Cells (5 × 10^5^ cells) from each experimental group (OP and CON *n* = 8 for each) were seeded in 48-well plates and allowed to adhere for 30 min at 37°C following stimulation or not with PMA 50 ng/mL for up to 3 h at 37°C. The antimicrobial activity of MPO was determined indirectly by catalysis of the production of hypoalous acids, such as hypochlorous acid (HOCl), from hydrogen peroxide (H_2_O_2_) and chloride ion (Cl^−^).

The MPO activity was not affected by the feeding strategy (*P* = 0.1270) and the interaction between *in vitro* PMA stimulation and the feeding strategy (*P* = 0.4385, Figure 3). However, PMA stimulation significantly increased the MPO activity (*P* = 0.0311). Furthermore, a positive correlation was found in OP neutrophils between the NE-DNA complexes produced by NETosis and MPO activity (*P* = 0.008, *r* = 0.97).

### Immunofluorescence Staining of NETs

**Figure 4:**
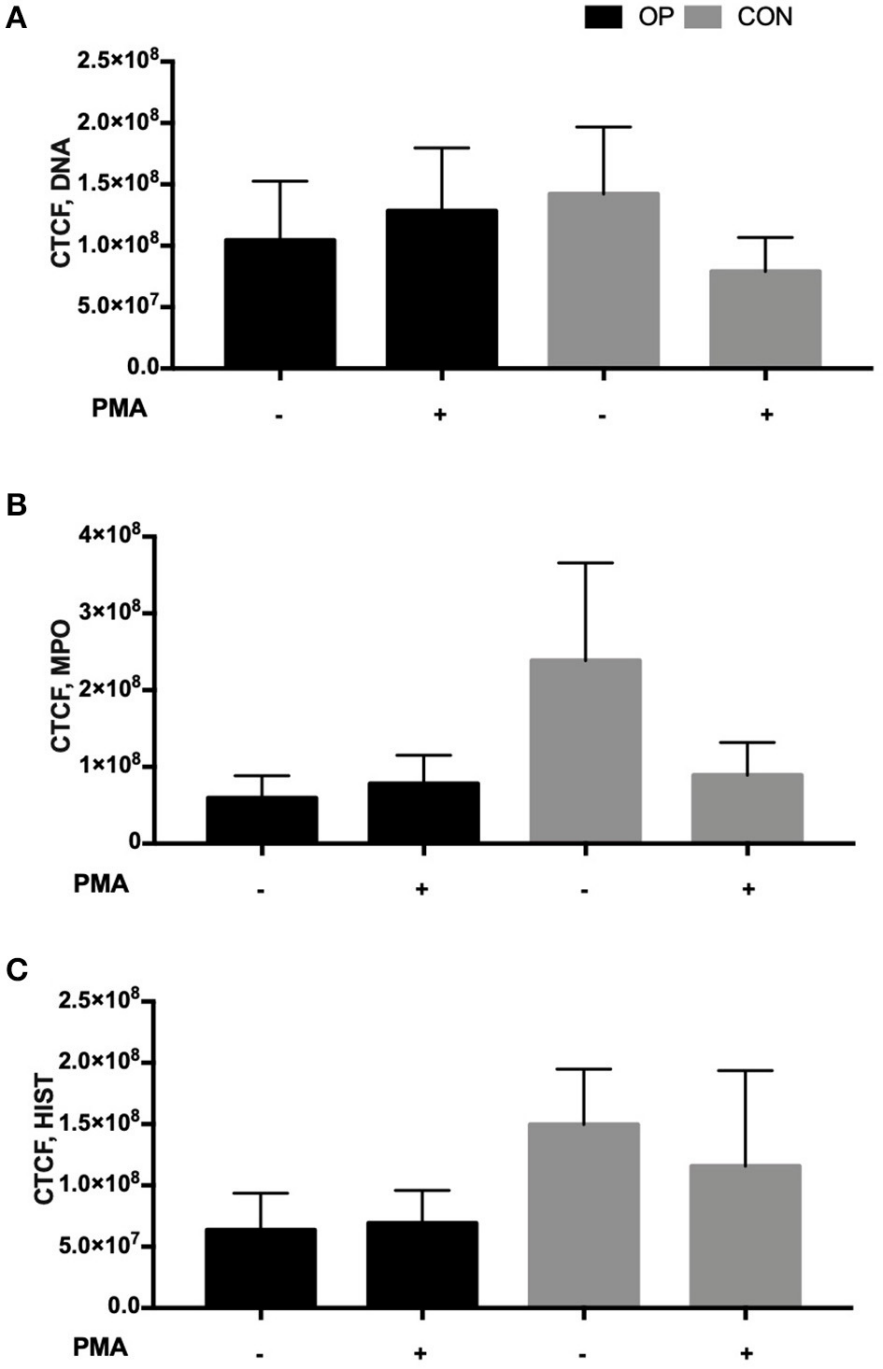
Mean of corrected total cell fluorescence (CTCF) = integrated density—(area of selected cell × mean fluorescence of background) ± of each experimental group (OP and CON, *n* = 4 for each) calculated using Image J software of **(A)** DNA, **(B)** MPO, and **(C)** Histone-H1 images. The mean fluorescence of the background was collected using areas without fluorescence adjacent to cells.

**Figure 5:**
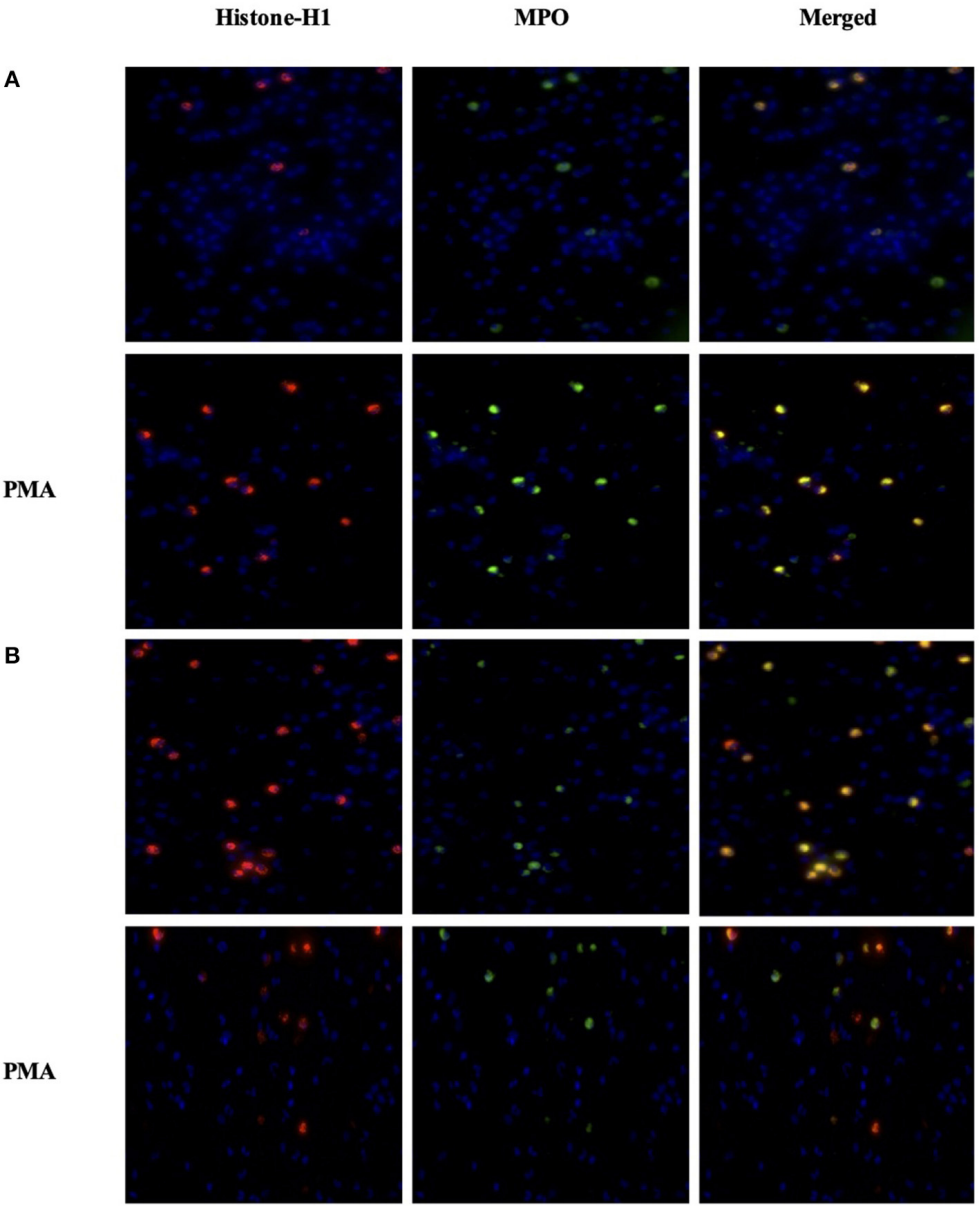
Imaging of neutrophils from the OP group (*n* = 4) **(A)** and from the CON group (*n* = 4) **(B)**. Cells (5 × 10^5^ cells) were seeded in 48-well plates and allowed to adhere for 30 min at 37°C. Cells were stimulated or not with PMA 50 ng/mL for up to 3 h. Cells were fixed in 4% paraformaldehyde for 15 min at RT and then permeabilized with 500 μl of Triton-x 0.5% in PBS for 20 min at RT. To detect Histone-H1 and MPO, each well was incubated with 5 μl (400 μl) Histone-H1 Polyclonal Antibody (Invitrogen) and 5 μl of Anti-Human Myeloperoxidase (MPO)-FITC overnight at 4°C. On the next day, the plate was washed twice with BSA 3%/PBS, then 500 μl of Goat anti-Rabbit IgG (H+L) Alexa Fluor plus 594 conjugated, as secondary antibody for Histone-H1, and Hoechst 33342 for nuclei detection. The plate imaged on Life Technologies EVOS™ XL Imaging System (Milan, Italy). The nucleus was detected by the DAPI channel, the MPO in the GFP channel, and the Histone-H1 in the Texas red channel.

To assess NETosis, neutrophils were stained with Anti-Human MPO, Histone-H1, and DAPI after *in vitro* PMA stimulation in order to depict a concomitant peptidylarginine deiminase 4 (PAD4)-dependent citrullination of histones that induces decondensation of DNA resulting in a mixture of DNA and bactericidal proteins, including MPO and NE, which are contained originally in intracytoplasmic granules. The images were collected and analyzed using ImageJ software as described in the methods. Data on CTCF did not show a significant difference in NETs between the CON and OP groups and after *in vitro* PMA stimulation (Figure 4). However, the images related to CRCF revealed a lower NET activation in the CON group after *in vitro* PMA stimulation (Figure 5).

## Discussion

In the present study, the response of peripheral neutrophils was investigated by an *ex vivo* trial focusing on the NET formation and MPO activity from *in vivo* dairy cows fed with olive pomace. Our results demonstrated that supplementing diet with OP (6%) supports the NETotic response of peripheral neutrophils when activated with PMA stimulation; on the contrary, the CON diet showed a pre-activated peripheral neutrophil without an additional response after PMA stimulation.

Ruminant farming has been threatened and impacted recently as a result of lacking knowledge on invading pathogens and host responses. One of the main contributing factors to health disorders is the uncontrolled inflammation activated by an altered bovine immune mechanism. These disorders are on the basis of several economically important infectious and metabolic diseases including mastitis, retained placenta, metritis, displaced abomasum, and ketosis (19). During inflammatory cascade, a complex biological response of both cellular, such as an increased movement of leukocytes and plasma components from the blood into inflamed tissues, and soluble factors, such as local antimicrobial factors, occurs (45, 46). The objective of host inflammatory responses is firstly removing the cause of tissue injury, restoring immune homeostasis, and returning tissues to normal function. The first cell type recognized during the early stage of inflammation are neutrophils, which are involved in adhering to the local endothelium more proximate to the site of infection. Neutrophils' migration occurs very rapidly, and their number increased within affected tissues as soon as 30–60 min following injury (47). Bovine neutrophils accounted for only 25% of total blood leukocyte numbers; however, it has been stated that mature lactating Holstein cows have a pool of more than 100 billion circulating neutrophils (48). It is interesting that the nature of neutrophil populations responding to a particular mammary stimulus depends on the intensity of the stimulus and the strength of the chemotactic agent (49). Once neutrophils became activated, they can generate NETs consisting of a network of fibers composed of chromatin and serine proteases, which are involved in trapping and killing bacteria. It has been found that after *in vitro* PMA stimulation, human neutrophils experience morphological changes among which include chromatin decondensation, loss of nuclear envelope, mixing of nuclear contents and cytoplasmic granular proteins, loss of membrane integrity, and release of cell free DNA (cfDNA) (3). Moreover, studies in murine, human, and canine neutrophils established that, during NETosis, the release of histones and DNA is activated by histone post-translational modification (50–52). In particular, histone citrullination, catalyzed by the enzyme peptidylarginine deiminase 4 (PAD4), causes the loss of positive charge of histones, which, in turn, destroys electrostatic interactions between DNA and histones causing chromatin decondensation and release of cfDNA during NETosis (53–55). Using immunofluorescence imaging, studies confirmed the presence of histone proteins on the NETs, including the core histones (H2A, H2B, H3, H4), linker Histone-H1, and the H2A–H2B–DNA complex (56). In a study by Papayannopoulos et al. (57), they suggested that H1 may have to be degraded first to allow for the subsequent degradation of core histones; indeed, when nuclei was pre-treated or coincubated with H1, MPO failed to partition with the nuclear fraction, which indicates that chromatin is the primary binding site of MPO *in vitro*. Therefore, NE and MPO require access to the core histones to degrade them and induce decondensation.

In the present study, the NETosis was evaluated using two different techniques: the ELISA and immunofluorescence imaging. In the first, the NE-DNA complexes were evaluated; in the second, the NETosis was checked by a colocalization of DNA, Histone-H1, and MPO in granules of neutrophils, aimed at a better understanding of this phenomenon. Data from both analyses suggested that OP dietary supplementation sustains neutrophil response after PMA stimulation. Conversely, neutrophils from the CON diet did not respond after PMA stimulation, being in a kind of pre-activated state, supporting the hypothesis of an impairment of neutrophil immune response with an increase in exposition to disease.

Bovine neutrophils actively participate in the inflammatory process of mastitis being responsible for the production of a wide variety of neutrophil β-defensins to fight invasive pathogens (58). Nevertheless, these antimicrobial molecules also damage the fragile inner layer of the mammary gland leading to a permanent tissue damage and decreasing the mammary epithelial cell participation in lactation. The MPO enzyme is produced in neutrophil primary granules that, in a system with hydrogen peroxide and halide, trigger an antimicrobial effect oxygen-dependent on pathogens both at the extracellular and intracellular levels (59). The quantitative analysis of MPO in milk can be used as a diagnostic marker to detect intramammary infections in dairy cattle (60). Results from the present study demonstrated that PMA stimulation increased the MPO activity of neutrophils as a result of their enhanced antimicrobial role when supplementation with OP was offered to cows.

Recently, a great number of therapies for livestock diseases have been promoted starting from natural byproducts, among which phytochemicals have attracted scientific attention for their anti-inflammatory activity and modulatory biological responses, principally orientated to treat or prevent microbial invasion as alternatives to antibiotics (61–63). *In vitro* co-cultured neutrophils with cold pressed terpene less Valencia orange oil increased their chemotaxis without altering their phagocytic ability and expressing a downregulation of pro-inflammatory immune response, resulting in the inhibition of bacterial growth without negatively altering the neutrophil function (64). Moreover, butyric acid, a short-chain fatty acid (SCFA), was found to exert potent anti-inflammatory action, activating neutrophils, inducing platelet activating factor (PAF), increasing CD63 expression, producing the release of matrix metalloproteinase-9 (MMP-9) and lactoferrin, and activating NET formation (65). Studies on SCFA-based pathways demonstrated that the production of SCFA is involved in subacute rumen acidosis and the activation of the inflammatory response (66). Similarly, NEFAs, among which OA is the principal released during lipomobilization at the time of calving in cows (67), have been suggested to have a potential role in innate immune responses or in the inflammatory process at parturition, when cows experience higher risk of contracting infectious diseases (68). In particular, OA was found to activate the bovine PMN responses *in vitro* by inducing intracellular calcium mobilization, MAPK phosphorylation, superoxide production, and release of granules containing CD11b and MMP-9 (69–71). Accordingly, our results demonstrated that OP dietary supplementation succeeded in making neutrophils more reactive to PMA activation by inducing NET formation and increasing the MPO activity. These results were confirmed by the positive correlation found between NE-DNA complexes produced during NETosis and intracellular H_2_O_2_ controlled by MPO activity, and were probably due to the major content of OA in the OP diet found in the present study. Neofytou et al. (72), supplementing cows with 10% of ensiled olive cake, with a level of OA of about 30% of the dietary lipids, observed a reduction of medium-chain FA concentration and increased long-chain FA (LCFA) levels in cow's milk (72). The direct transfer between blood to milk constituent (73) could suggest that OA from the diet could have represented a suitable energy source to initiate an effective neutrophil response. In support of this hypothesis, previous studies demonstrated that n-3 FA supplementation of dairy cows exposed to high ambient temperatures attenuated the alterations of lymphocyte proliferation during negative energy balance and enhanced immune responses (74, 75).

## Conclusions

Data from the present study demonstrated that supplementation of 6% OP in the diet of dairy cow supports the NETosis after PMA stimulation with a concomitant increase in the MPO activity. These findings were corroborated by the colocalization of citrullination of Histone-H1 and MPO expression of neutrophils by immunofluorescence imaging. A comprehensive understanding of the linkages between potentiality of nutrients and the first line of cellular immunity defense will be useful in planning nutritional regimes and reduce disease susceptibility in lactating cows. At present, the crucial role of neutrophils has emerged in both antimicrobial and the newest immunomodulatory properties at the crossroad of innate and adaptive immunity. Studies on bovine neutrophils are needed to better understand the mechanisms of their modulation and regulatory function pointed out to control various health disorders at the time of immunosuppression as a diagnostic tool for improving the productivity of dairy cows.

## Data Availability Statement

The raw data supporting the conclusions of this article will be made available by the authors, without undue reservation.

## Ethics Statement

The animal study was reviewed and approved by Ethics Committee for animal testing–CES, University of Foggia.

## Author Contributions

MC and MGC were involved in the original design of the study. SC was responsible for the on-farm trial. MGC was responsible for analytical procedures, RM and AS analyzed the data, and MC, MGC, and MA wrote the manuscript. All authors have read, revised critically, approved the final version of the manuscript, and contributed to the preparation of the manuscript.

## Conflict of Interest

The authors declare that the research was conducted in the absence of any commercial or financial relationships that could be construed as a potential conflict of interest.
