# Supplementary material for: NETosis of Peripheral Neutrophils Isolated From Dairy Cows Fed Olive Pomace
Source: Front Vet Sci. 2021 Apr 29;8:626314. doi: 10.3389/fvets.2021.626314 (PMC8118642; doi:10.3389/fvets.2021.626314)
Supplement: Supplementary file 1 [file Data_Sheet_1.docx]

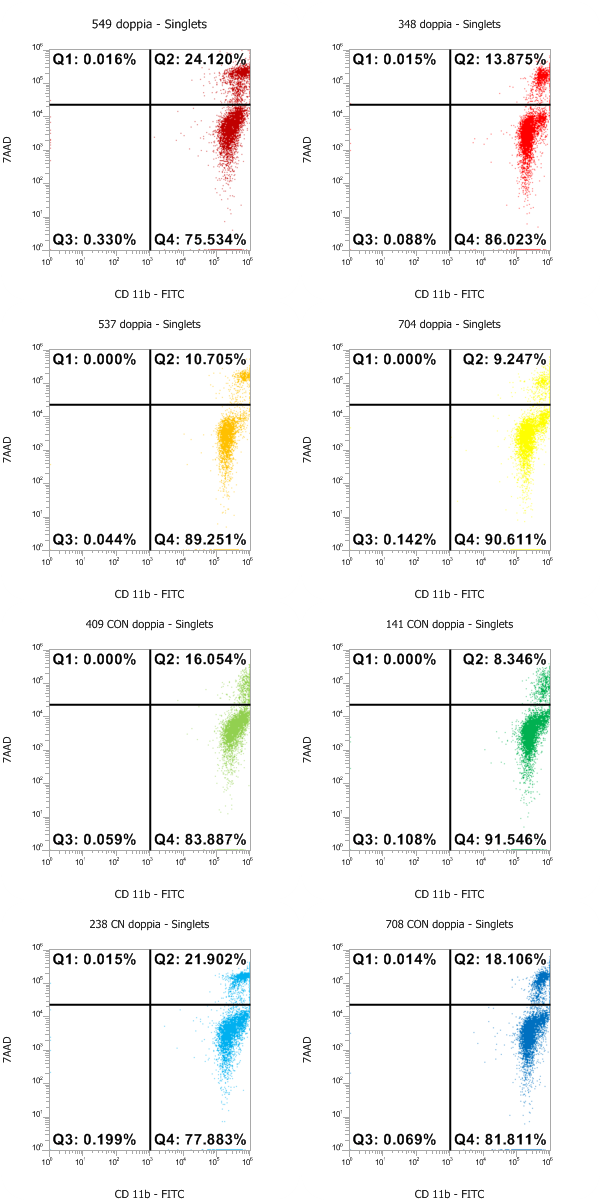


**h)**

**g)**

**f)**

**e)**

**d)**

**c)**

**b)**

**a)**

**Supplementary Figure 1** Overlay of Dot plot of CD11b and 7-AAD; a,b,c,d for OP group, e,f,g,h for CON group, respectively.
